# CYP2D6 gene variants: association with breast cancer specific survival in a cohort of breast cancer patients from the United Kingdom treated with adjuvant tamoxifen

**DOI:** 10.1186/bcr2629

**Published:** 2010-08-23

**Authors:** Jean E Abraham, Mel J Maranian, Kristy E Driver, Radka Platte, Bolot Kalmyrzaev, Caroline Baynes, Craig Luccarini, Mitul Shah, Susan Ingle, David Greenberg, Helena M Earl, Alison M Dunning, Paul DP Pharoah, Carlos Caldas

**Affiliations:** 1 Department of Oncology, Strangeways Research Laboratory, University of Cambridge, 2 Worts Causeway, Cambridge, CB1 8RN, UK; 2 Cambridge Breast Unit and NIHR Cambridge Biomedical Research Centre, University of Cambridge NHS Foundation Hospitals, Hills Road, Cambridge, CB2 0QQ, UK; 3 Cancer Research UK Cambridge Research Institute, Li Ka Shing Centre, Robinson Way, Cambridge, CB2 0RE, UK; 4 Eastern Cancer Registration and Information Centre, Unit C-Magog Court, Shelford Bottom, Hinton Way, Cambridge, CB22 3AD, UK

## Abstract

**Introduction:**

Tamoxifen is one of the most effective adjuvant breast cancer therapies available. Its metabolism involves the phase I enzyme, cytochrome P4502D6 (CYP2D6), encoded by the highly polymorphic *CYP2D6 *gene. *CYP2D6 *variants resulting in poor metabolism of tamoxifen are hypothesised to reduce its efficacy. An FDA-approved pre-treatment *CYP2D6 *gene testing assay is available. However, evidence from published studies evaluating *CYP2D6 *variants as predictive factors of tamoxifen efficacy and clinical outcome are conflicting, querying the clinical utility of *CYP2D6 *testing. We investigated the association of *CYP2D6 *variants with breast cancer specific survival (BCSS) in breast cancer patients receiving tamoxifen.

**Methods:**

This was a population based case-cohort study. We genotyped known functional variants (*n *= 7; minor allele frequency (MAF) > 0.01) and single nucleotide polymorphisms (SNPs) (*n *= 5; MAF > 0.05) tagging all known common variants (tagSNPs), in *CYP2D6 *in 6640 DNA samples from patients with invasive breast cancer from SEARCH (Studies of Epidemiology and Risk factors in Cancer Heredity); 3155 cases had received tamoxifen therapy. There were 312 deaths from breast cancer, in the tamoxifen treated patients, with over 18000 years of cumulative follow-up. The association between genotype and BCSS was evaluated using Cox proportional hazards regression analysis.

**Results:**

In tamoxifen treated patients, there was weak evidence that the poor-metaboliser variant, CYP2D6*6 (MAF = 0.01), was associated with decreased BCSS (*P *= 0.02; HR = 1.95; 95% CI = 1.12-3.40). No other variants, including CYP2D6*4 (MAF = 0.20), previously reported to be associated with poorer clinical outcomes, were associated with differences in BCSS, in either the tamoxifen or non-tamoxifen groups.

**Conclusions:**

CYP2D6*6 may affect BCSS in tamoxifen-treated patients. However, the absence of an association with survival in more frequent variants, including CYP2D6*4, questions the validity of the reported association between *CYP2D6 *genotype and treatment response in breast cancer. Until larger, prospective studies confirming any associations are available, routine *CYP2D6 *genetic testing should not be used in the clinical setting.

## Introduction

Tamoxifen has been the standard treatment for oestrogen receptor (ER)-positive breast cancer for more than three decades. Indications for its use [1] include: metastatic disease in women (pre- and post-menopausal) and men; adjuvant therapy in pre- and post-menopausal women with breast cancer (lymph node positive and negative); preventative therapy in women at high risk of breast cancer; ductal carcinoma *in situ *post-resection; and for the prevention of contra-lateral breast cancer. There are proven benefits associated with five years of tamoxifen treatment in ER-positive breast cancer patients. There is a significant decrease in the annual recurrence rate, improved overall survival (OS) and a reduction of the breast cancer mortality rate by a third [2].

**Figure 1:**
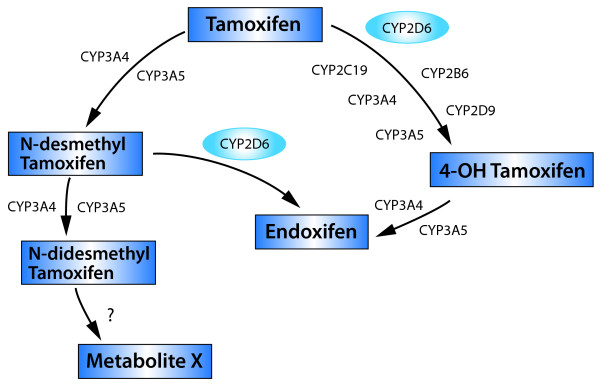
**Tamoxifen metabolic pathway**. CYP2D6, Cytochrome P450 2D6; CYP2D9, Cytochrome P450 2D9; CYP3A4, Cytochrome P450 3A4; CYP3A5, Cytochrome P450 3A5; CYP2B6, Cytochrome P450 2B6; CYP2C19, Cytochrome P450 2C19.

Tamoxifen is extensively metabolised after oral administration (Figure 1). N-desmethyl tamoxifen, the major metabolite found in patients' plasma, undergoes secondary metabolism to 4-hydroxy-N-desmethyl tamoxifen (endoxifen). The enzyme involved in this conversion is cytochrome P450 2D6 (CYP2D6), which also converts tamoxifen to 4-hydroxy tamoxifen. This metabolite undergoes secondary metabolism to endoxifen. It is widely accepted that the majority of the anti-proliferative effect of tamoxifen occurs via its active metabolites [3-5]. 4-hydroxy tamoxifen has at least 100-fold greater affinity for the ER than tamoxifen, and has a similarly increased potency in anti-proliferative action. Endoxifen has an equivalent anti-proliferative potency and ER binding ability to 4-hydroxy tamoxifen [6-8] but is present in higher concentrations in the plasma.

Any factor that diminishes production of these metabolites could impact on tamoxifen efficacy. Several enzymes are involved in these metabolic pathways, with *CYP2D6 *playing a pivotal role [9]. *CYP2D6 *is a polymorphic gene with over 90 documented alleles [10]. Some of these variants are associated with either reduced or absent *CYP2D6 *enzyme activity. Pharmacokinetic work using probe drugs such as debrisoquine [11], first demonstrated the effects of *CYP2D6 *variants on drug metaboliser status. CYP2D6 metaboliser function is generally categorised into four groups: poor-metaboliser (PM); intermediate-metaboliser (IM); extensive-metaboliser (EM) and ultra-metaboliser (UM) [12]. It has been hypothesised that patients with PM and IM phenotypes generate reduced plasma concentrations of active metabolites from a standard tamoxifen dose, hence reducing its efficacy. Several studies have explored the correlation between *CYP2D6 *genotype, and either plasma metabolite levels and/or clinical outcome in patients treated with tamoxifen. Ten studies have demonstrated an association between putative PM variants of *CYP2D6 *and poorer clinical outcome [13-22]. However other studies either found no such association or the opposite results [23-26]. These studies have been heterogeneous in both design and analytic methodology. After reviewing five of these conflicting studies, Lash and colleagues [27,28] concluded that the most straightforward explanation for the conflicting results is that the null hypothesis cannot be rejected. The majority of these studies use disease free survival (DFS) or progression free survival (PFS) as the clinical endpoints, but there is considerable doubt about whether these are the most valid endpoints, particularly in the adjuvant setting where breast cancer specific survival (BCSS) or distant disease free survival may be better endpoints [29]. Our study primarily uses BCSS as the endpoint although OS was also assessed.

The CYP2D locus contains three highly homologous sequences of which *CYP2D6 *is the functional gene, while *CYP2D7 *and *CYP2D8 *are non-functional pseudogenes [30,12]. *CYP2D6 *shares 93% sequence similarity with *CYP2D7 *and 89% with *CYP2D8 *(BLAST alignment). This degree of homology can reduce the specificity of genotyping TaqMan assays for *CYP2D6*, leading to unreliable genotype classifications and potentially unreliable clinical associations. Hosking and colleagues [30] draw particular attention to this problem in CYP2D6 in their article on detection of genotyping errors by testing for deviation from Hardy-Weinberg equilibrium (HWE *P *< 0.05) and such testing is now a well established part of genotyping quality control [31,32]. Technical reasons such as assay non-specificity can impact on the distribution of genotypes for any one variant. When a variant deviates from HWE, the significance of any association made is potentially unreliable.

Punglia and colleagues [33] performed a modelling analysis to investigate whether women with EM genotypes of *CYP2D6 *might have improved outcomes when treated with tamoxifen rather than an aromatase inhibitor (AI). They concluded that women carrying the EM variants might have lower relapse rates when treated with tamoxifen. Such modeling has limitations [34], but if correct, *CYP2D6 *genotype would clearly influence the decision to use either tamoxifen or AIs.

The Food and Drug Administration (FDA) approved the diagnostic tool, the Amplichip^® ^CYP450 Test, which screens for 29 variants in the *CYP2D6 *gene and two in *CYP2C19 *[35]. Prior to such a test being routinely implemented in clinical practice, it is important that large studies, with rigorous genotyping quality control are used to assess the magnitude, if any, of the association between *CYP2D6 *variants and valid clinical endpoints including BCSS and OS. Currently, such studies have not been published that establish the clinical utility of *CYP2D6 *genotyping in determining treatment choice or dose, in relation to tamoxifen therapy.

We have used two approaches to investigate the relation between germline variation in the *CYP2D6 *gene and BCSS and OS in breast cancer patients treated with tamoxifen. We evaluated the effect of single nucleotide polymorphisms (SNPs) representing known functional variants (PM, IM and UM; minor allele frequency (MAF) > 0.01) on clinical outcome after adjuvant tamoxifen therapy. We also carried out an empirical evaluation of common genetic variation in *CYP2D6 *and clinical outcome using a standard SNP tagging approach that captures common variation without being dependent on any prior knowledge of function.

## Materials and methods

### Study population

Breast cancer cases were drawn from Studies of Epidemiology and Risk factors in Cancer Heredity (SEARCH), an ongoing population-based case-control series, with cases ascertained through the Eastern Cancer Registration and Information Centre (ECRIC) in England, a regional population-based cancer registry. All women diagnosed with invasive breast cancer before the age of 55 years since 1991 and still alive in 1996 (prevalent cases, median age 48 years), together with all those diagnosed before the age of 70 years between 1996 and the present (incident cases, median age 54 years) were eligible to take part. All patients were non-metastatic at recruitment. Questionnaires and a blood kit were sent to eligible patients who had consented to participate. The categories of information collected from the questionnaires included personal information, reproductive history, medical history, drug/treatment history and family history. Of those eligible, 67% returned questionnaires (6,951) and 64% (6,640) of those eligible provided a blood sample for DNA analysis. Data on tumour morphology, grade, and stage were obtained from the medical record. Eligible patients who did not take part were similar to responders for age at diagnosis, histopathological morphology and grade, but the proportion of clinical stage III/IV cases was higher in non-participants. The total number of cases available for analysis was 6,640; 5,349 (81%) incident cases and 1,291 (19%) prevalent cases. The study is approved by the Eastern Region Multicentre Research Ethics Committee. All participants provided written informed consent.

Information on the use of adjuvant hormone therapy is obtained from the medical records by ECRIC, but these data do not specify the drug used. We therefore used the self-reported questionnaire data to identify those women who had taken adjuvant tamoxifen. Within the East Anglia/Cambridgeshire region tamoxifen was the first-line adjuvant treatment until 2006, except in the case of high risk, lymph node-positive patients, where AIs have been in use since 2005. The standard tamoxifen dose prescribed is 20 mg once daily. Of the eligible cases, 3,155 confirmed treatment with tamoxifen and 3,485 patients either did not receive any adjuvant hormone therapy or they did not report what type of hormone therapy they had taken. In order to validate the questionnaire data, we checked the medical records of 120 cases for treatment history and compared this with questionnaire responses. The concordance rate between self-reported treatment and treatment recorded within the medical notes was 100%. Table 1 details the characteristics of the study participants.

**Table 1:** Breast cancer patient characteristics

		Patients WITH self-reported tamoxifen treatment *n *= 3155^‡^	^†^Patients WITHOUT self-reported tamoxifen treatment *n *= 3485^‡^
**Median age**		53	53
**Age range**		24-69	23-69

**Tumour size group (cm)**	*N/A	913 (13.8%)	1,275 (19.2%)
	**< 2**	1,448 (21.8%)	1,380 (20.8%)
	**2-5**	710 (10.7%)	751 (11.3%)
	**5+**	84 (1.3%)	79 (1.2%)

**Grade**	**1**	670 (10.1%)	599 (9%)
	**2**	1,487 (22.4%)	1,318 (19.8%)
	**3**	560 (8.4%)	1,019 (15.3%)
	*N/A	438 (6.6%)	549 (8.3%)

**Positive nodes**	**0**	1,606 (24.2%)	1,476 (22.2%)
	**1-3**	603 (9.1%)	591 (8.9%)
	**4-9**	140 (2.1%)	200 (3%)
	**10+**	57 (0.9%)	78 (1.2%)
	*N/A	749 (11.3%)	1,140 (17.2%)

**Stage**	**1**	1,624 (24.5%)	1,655 (24.9%)
	**2**	1,359 (20.5%)	1,505 (22.7%)
	**3**	99 (1.5%)	146 (2.2%)
	**4**	28 (0.4%)	50 (0.8%)
	*N/A	45 (0.7%)	129 (1.9%)

**ER**	*N/A	959 (14.4%)	1,117 (16.8%)
(oestrogen receptor status)	**Negative**	207 (3%)	668 (10.1%)
	**Positive**	1,989 (30%)	**^§^**1,699 (25.6%)

**Surgery**	**Yes**	1,982 (29.8%)	1,860 (28%)
	**No**	132 (2%)	220 (3.3%)
	*N/A	1,041 (15.7%)	1,405 (21.2%)

**Chemotherapy**	**Yes**	587 (8.8%)	782 (11.8%)
	**No**	1,527 (23%)	1,298 (19.5%)
	*N/A	1,041 (15.7%)	1,405 (21.2%)

**Person years follow-up**		18,860.38	19,189.94
^a^**Number of events**		312	418

*N/A - Data not available.

§Of 1,699 patients, 1,119 (16.9%) patients received hormone treatment but did not document which type; 3 (0.05%) had combined tamoxifen and aromatase treatment; 232 (3.5%) received aromatase; 341(5.1%) received no hormone treatment; 4 (0.06%) patients had no data available.

†Some patients in this group stated that they received adjuvant hormone treatment but did not document which drug.

‡Percentages given are from total of 6,640.

^a^Breast cancer specific deaths.

### Concomitant medication

It has been postulated that concomitant use of drugs that inhibit CYP2D6 function may also affect tamoxifen efficacy and clinical outcome [7]. As part of the questionnaire, patients were requested to record current medication at the time of recruitment. CYP2D6 inhibitor (e.g. fluoxetine, cimetidine) use was recorded in 460 patients [see Supplementary table S1 in Additional file 1], of whom 193 cases had received adjuvant tamoxifen. However, we cannot exclude use of such medication pre-recruitment, and in addition we do not have details of duration of treatment with CYP2D6 inhibitors.

#### Clinical follow-up

The ECRIC used active follow-up at three and five years after diagnosis and then at five-year intervals until the end of 2005. Follow-up information and OS (all-cause-mortality) were obtained by searching hospital information systems for recent visits. If a patient had not had a recent visit, the patient's general practitioner was contacted to obtain the vital status. Continual, passive follow-up is also carried out by death certificate flagging through the Office of National Statistics. Since 2006, the National Health Strategic Tracing Service has been actively searched to determine the vital status of these patients. The most recent search was on 31st July 2008. The lag time with this process is a few weeks for cancer deaths and two months to a year for non-cancer deaths and so follow-up was censored six months before the most recent search. Breast cancer specific mortality was defined as a death in which breast cancer was given as the cause of death on Part I of the death certificate. All-cause mortality was defined as death from any cause.

### Selection of functional SNPs and haplotypes

Many rare variants of *CYP2D6 *have been reported and their nomenclature is complex [36]. Some of these variants are single nucleotide changes and others are haplotypes - alleles at multiple loci that are inherited together. An extensive review of the literature identified 15 putative functional variants [37-42] that occur in populations of European origin, of which seven of these were successfully genotyped [see Supplementary table S2 in Additional file 1]: CYP2D6*1; CYP2D6*4; CYP2D6*5; CYP2D6*6b/c; CYP2D6*9; CYP2D6*10; CYP2D6*41; CYP2D6*UM (UM refers to ultra-metaboliser phenotype).

The CYP2D6*6 allele is defined by a frame-shift mutation protein truncating deletion -1707del*t*. However, we were unable to design a Taqman assay for this variant and could not genotype it directly. This allele is sub-divided into four sub-types: CYP2D6*6a; CYP2D6*6b; CYP2D6*6c; and CYP2D6*6d. CYP2D6*6b and CYP2D6*6c carry the 1976*g*>*a *variant, which we successfully genotyped. There is little data in the literature to determine the proportion of all CYP2D6*6 alleles that are accounted for by CYP2D6*6b and CYP2D6*6c, but any misclassification of CYP2D6*6 will be very small as all the alleles are rare.

The association between individual PM variants (CYP2D6*4; CYP2D6*5; CYP2D6*6) and BCSS and OS was assessed. Similarly, the association between individual IM variants (CYP2D6*41; CYP2D6*9; CYP2D6*10) and BCSS and OS were assessed. In addition, two PM/IM combined models were also assessed for any association with BCSS and OS. The unadjusted and adjusted results, as well as the variables adjusted for are given in Table 2. The PM/IM group are classified as carriers of two variants (i.e. rare homozygote alleles) for at least one of the SNPs associated with PM or IM variants as stated previously. The PM/IM model 1 compares the PM/IM group with individuals who carried two copies of the wild-type (EM) allele at all SNPs (i.e. common homozygotes) or individuals who carried a single variant allele at a single SNP (i.e. heterozygotes). PM/IM model 2 compares individuals who carried two copies of the wild-type allele (EM) at all SNPs (i.e. common homozygotes).

**Table 2:** Results of unadjusted and adjusted Cox regression analysis for breast cancer specific survival in common (tagSNPs) and functional polymorphisms of CYP2D6

			No tamoxifen (unadjusted)			All tamoxifen-treated patients (unadjusted)			ER-positive patients treated with tamoxifen (unadjusted)			ER-positive patients treated with tamoxifen (adjusted)		
						
†SNP	Metaboliser Status	MAF**	*P*-value	Hazard ratio	95% CI**	*P*-value	Hazard ratio	95% CI**	*P*-value	Hazard ratio	95% CI**	*P*-value	Hazard ratio	95% CI**
**CYP2D6 functional SNPs**														
CYP2D6*41	(IM)	0.09	0.89	1.02	0.72-1.46	0.12	0.79	0.59-1.07	0.43	0.85	0.57-1.27	0.69	0.88	0.48-1.62
CYP2D6*4	(PM)	0.2	0.34	0.88	0.69-1.15	0.89	1.01	0.83-1.24	0.93	1.01	0.78-1.32	0.39	1.17	0.82-1.68
CYP2D6*5	(PM)	0.04	0.49	2.01	0.28-14.3	^†^n/a	^†^n/a	^†^n/a	^†^n/a	^†^n/a	^†^n/a	^†^n/a	^†^n/a	^†^n/a
^‡^CYP2D6*6	(PM)	0.01	0.76	1.17	0.43-3.14	0.02	1.95	1.12-3.40	0.04	2.14	1.05-4.36	0.33	1.8	0.56-5.80
CYP2D6*9	(IM)	0.03	0.53	0.8	0.40-1.60	0.45	1.18	0.76-1.83	0.62	1.18	0.62-2.23	0.33	1.52	0.65-3.52
CYP2D6*10	(IM)	0.02	0.93	0.96	0.42-2.19	0.61	1.22	0.57-2.59	^†^n/a	^†^n/a	^†^n/a	^†^n/a	^†^n/a	^†^n/a
CYP2D6*UM	(UM)	0.08	0.65	1.57	0.22-11.3	0.45	0.47	0.07-3.34	^†^n/a	^†^n/a	^†^n/a	^†^n/a	^†^n/a	^†^n/a
^§^CYP2D6*PM Model 1	(PM)	^‡‡^N/A	0.46	0.77	0.39-1.52	0.78	0.93	0.55-1.57	0.98	1.01	0.51-2.00	0.32	1.57	0.64-3.84
^§§^CYP2D6*PM Model 2	(PM)	^‡‡^N/A	0.85	0.97	0.72-1.31	0.63	1.06	0.84-1.34	0.82	0.96	0.70-1.32	0.21	1.35	0.84-2.16
**TagSNPs**														
CYP2D6_01t	¹N/A	0.32	0.33	0.89	0.71-1.12	0.3	0.91	0.77-1.08	0.97	1	0.79-1.25	0.65	0.93	0.66-1.3
CYP2D6_02t	¹N/A	0.46	0.07	1.21	0.98-1.49	0.98	1	0.86-1.17	0.89	0.98	0.79-1.22	0.43	0.88	0.65-1.20
CYP2D6_03t	¹N/A	0.24	0.18	0.84	0.65-1.08	0.93	1.01	0.84-1.21	0.93	1.01	0.79-1.30	0.93	1.02	0.72-1.43
CYP2D6_04t	¹N/A	0.08	0.34	1.19	0.83-1.72	0.34	0.86	0.63-1.17	0.88	1.03	0.70-1.52	0.43	1.25	0.72-2.19
CYP2D6_05t	¹N/A	0.22	0.41	0.9	0.69-1.16	0.94	1.01	0.83-1.22	0.48	0.91	0.69-1.18	0.6	1.1	0.77-1.59

*Adjusted values in all cases, except PM models, include adjustment for grade, stage, chemotherapy, surgery, lymph node status, ER and tumour size.

** 95% CI, 95% confidence interval; ER, oestrogen receptor; MAF, mean allele frequency; SNP, single nucleotide polymorphisms.

^†^n/a, No calculations possible due to low sample number.

^§^The PM group are classified as carriers of two variant alleles (rare homozygote alleles) for at least one of the functional SNPs associated with PM or intermediate metaboliser (IM) status. Where IM = CYP2D6*41; CYP2D6*9; CYP2D6*10 and PM = CYP2D6*4; CYP2D6*5; CYP2D6*6. In CYP2D6*Poor Metaboliser (PM) Model 1: the PM group (classified as stated) is compared with individuals who carried two copies of the wild-type allele (EM) at all SNPs (i.e. common homozygotes) or individuals who carried a single variant allele at a single SNP (heterozygotes). Adjusted values in all cases include adjustment for grade, stage, chemotherapy, surgery, tumour size and ER status.

^§§^The PM group was classified as individuals carrying at least one variant allele at one or more of the functional SNPs (heterozygotes and rare homozygotes), associated with PM or intermediate metaboliser (IM) status. Where IM = CYP2D6*41; CYP2D6*9; CYP2D6*10 and PM = CYP2D6*4; CYP2D6*5; CYP2D6*6. In CYP2D6*Poor Metaboliser (PM) Model 2: the PM group (classified as stated) is compared with individuals who carried two copies of the wild-type allele (EM) at all SNPs (common homozygotes). Adjusted values in all cases include adjustment for grade, stage, chemotherapy, surgery, tumour size and ER status.

^‡‡ ^N/A, not applicable.

^‡^CYP2D6*6 refers to the 1976g > a variant, which is associated with CYP2D6*6b and CYP2D6*6c.

### Selection of tag SNPs

SNP tagging aims to identify a set of SNPs (tagSNPs; MAF > 0.05) that efficiently tags all the common variations in *CYP2D6 *with an estimated r^2 ^of more than 0.8. R^2 ^is the square of the correlation coefficient between a pair of SNPs. The loss in power incurred by using a marker SNP in place of a true causal SNP is directly related to r^2 ^value as effective sample size is directly proportional to r^2^. Where the common variation has not been systematically identified, a set of SNPs that tags the known common variation will also tag any hitherto unidentified SNPs in the gene with reasonable efficiency [43]. Data from the International HapMap Project [44] European samples of 30 parent-offspring trios were used to select tagSNPs. The 4.4 kb *CYP2D6 *gene is not well represented in any SNP databases so to obtain comprehensive coverage of the gene, a 100 kbp region surrounding the *CYP2D6 *gene was included. Five tagSNPs were selected using the aggressive 2- and 3-SNP tagging option in the Tagger programme implemented in Haploview. These five tagSNPs tagged 46 of the 47 common SNPs (98%) with r^2 ^of more than 0.8.

### Taqman genotyping

Genotyping was carried out using Taqman^® ^(Applied Biosystems Europe BV, UK Branch, Warrington, Cheshire, UK) according to manufacturer's instructions. Primers and FAM and VIC labelled probes were supplied directly by Applied Biosystems (UK Branch, Warrington, Cheshire, UK) as Assays-by-Design™. All assays were carried out in 384-well plates. Each plate included negative controls (with no DNA) and 12 randomly dispersed samples were selected from each 384-well plate and duplicated on a separate concordance plate. The original sample and its duplicate were then compared to ensure concordant genotype calls. Plates were read on the ABI Prism 7900 using the Sequence Detection Software (Applied Biosystems, UK Branch, Warrington, Cheshire, UK). Failed genotypes were not repeated. Assays in which the genotypes of duplicate samples did not show more than 95% concordance were discarded and replaced with alternative assays with the same tagging properties. Call rates for each assay were above 95%.

### Nested PCR genotyping

Initial attempts to genotype CYP2D6*4, with a specifically designed TaqMan assay and genomic DNA resulted in poor segregation of genotypes on cluster plots with overlapping clusters and errors in genotype calls. The distribution of genotypes deviated significantly from those expected under HWE. Direct sequencing of selected DNA samples confirmed those errors in TaqMan genotyping calls. These errors are likely to be due to the presence of the pseudogenes.

To increase assay specificity, we used a nested PCR approach for CYP2D6*4 to pre-amplify *CYP2D6*-specific template DNA prior to a standard TaqMan assay. We designed a pair of primers flanking SNP CYP2D6*4 on both sides with sequences highly specific and unique for the *CYP2D6 *gene (*CYPD6 *- Forward primer (F1): 5'GCATAGGGTTGGAGTGGGT3'; Reverse primer (R3): 5'TCCTCGGTCTCTCGCTCCG3' [see Supplementary figure S1, sequence alignment, in Additional file 2]. All genomic DNA samples were amplified using this primer pair under the following PCR conditions: 1 × AmpliTaq Gold^® ^Buffer II (ABI, Applied Biosystems Europe BV, UK Branch, Warrington, Cheshire, UK), 2 mM MgCl_2_, 0.05 U/ul AmpliTaq Gold DNA Polymerase (ABI, Applied Biosystems Europe BV, UK Branch, Warrington, Cheshire, UK), 0.25 mM dNTPs mix, 0.5 uM of each primer, 1 ng/ul genomic DNA. PCR program: 1) 95°C for 10 minutes; 2) 40 cycles of 94°C for 30 seconds, 66°C for 30 seconds, 72°C for 45 seconds; 3) 72°C for 10 minutes; 4) storage at 4°C. Aliquots of 5 × diluted PCR products were used as a template DNA for genotyping with CYP2D6*4 TaqMan Assay. Genotypes obtained were confirmed by direct sequencing of selected samples.

### Real-time PCR

The CYP2D6*5 (gene deletion) and CYP2D6*UM (gene duplication) variants were identified using a biplex Taqman real-time quantification assay as per the Schaeffeler and colleagues protocol [45]. Samples from patients with the known genotype CY2D6*1/*1 (EM or wild-type variant) were used to create the standard curve for the real-time PCR analysis. Also analysed on the same plate were samples with the known genotypes CY2D6*1/*5; CY2D6*5/*5; CY2D6*1/*2×*1. No known homozygote CYP2D6*UM (CY2D6*2×*1/*2×*1) sample was available. The samples with a homozygote deletion CY2D6*5/*5 were clearly identifiable; however, the range of expression values for CY2D6*1/*1, CY2D6*1/*5 and CY2D6*1/*2×*1 did not make it possible to distinguish between heterozygotes and common homozygotes. Homozygote CYP2D6*UM were recognised as lying more than two standard deviations from the wild-type and heterozygote clusters.

### Statistical methods

Cox regression analysis was used to test for an association between SNP genotype, BCSS and OS (all-cause mortality). The time at risk began on the date of diagnosis and time under observation began on the date of blood sample receipt and ended on the date of death from any cause, or, if death did not occur, on 31st January 2008. Thus, cases do not contribute to hazard estimation until they are under observation. This allows for the difference in ascertainment of incident and prevalent cases. Proportional hazards is a property of the true underlying biological model and not related to study design. A recent publication by Azzato and colleagues has demonstrated that the use of prevalent cases does not result in a bias of the hazard ratio estimate, provided the proportional hazards assumption is correct and the 'left truncation' (ascertainment/recruitment after diagnosis) is properly accounted for in the analysis [46]. Follow-up was censored at 10 years after diagnosis, because follow-up became less reliable for each individual after 10 years. The proportional hazards assumption was evaluated by visual inspection of log-log plots, as well as tested analytically using Schoenfeld residuals. We used a 1 degree of freedom (d.f.) trend test based on the number of rare alleles carried as the primary test of association, as the true underlying genetic model is not known. This test provides reasonable power for dominant or co-dominant genetic models, but limited power if the underlying model is recessive. For the rare, functional alleles, power to detect a recessive effect would be extremely limited as individuals who carry two copies of the minor allele are rare in the population. Supplementary table S3 in Additional file 1 shows the potential power of this study to detect a hazard ratio (HR) of 1.85. The HR per number of rare alleles carried, with associated 95% confidence limits, was estimated from the Cox regression. All analyses were performed in Intercooled Stata, version 10 (StataCorp LP, Texas, USA).

## Results

The five tagSNPs (MAF > 0.05) and seven putative functional SNPs (MAF > 0.01) were genotyped in 6,640 cases of predominantly Caucasian ethnicity (98.8%) [see Supplementary table S2 in Additional file 1]. Greater than 97% of samples were successfully genotyped for each SNP and the genotype distributions are shown in Supplementary table S4 in Additional file 1. The results of the association between each SNP and BCSS, according to tamoxifen treatment status are shown in Table 2. The results of the association between each SNP and OS are shown in Supplementary table S5 in Additional file 1. A summary of the results, independent of tamoxifen status, are given in Supplementary table S6 in Additional file 1. We have adhered to the REMARK [47] recommendations for reporting tumour marker studies as these criteria create a coherent and transparent framework for the reporting a wider range of study designs [see Supplementary table S7 in Additional file 3].

There was no statistically significant association between BCSS or OS and genotype for any of the tagSNPs for common variation at the *CYP2D6 *locus either in patients receiving or not receiving tamoxifen. There was no significant association between BCSS or OS and the putative functional alleles termed CYP2D6-*1; *4; *5; *9; *10; *41; *UM. Of particular note is the lack of association for the PM allele that has most commonly been reported to be associated with poorer clinical outcome in a Caucasian population - CYP2D6*4 (For BCSS: HR = 1.01; 95% confidence interval (CI) = 0.83 to 1.24; *P *= 0.89; and OS: HR = 1.06; 95% CI = 0.90 to 1.26; *P *= 0.5).

As patients may carry more than one variant allele and the power to detect any single variant is limited, we also classified individuals with at least one known functional allele as PM/IM and individuals with two normal alleles as EM. There was no difference in survival between these two groups (BCSS: HR = 0.93; 95% CI = 0.55 to 1.57; *P *= 0.78 and OS: HR = 0.98; 95% CI = 0.63 to 1.54; *P *= 0.94). Nor was there a difference between patients, carrying at least two variant alleles compared with EM patients or those who carried just a single variant (BCSS: HR = 1.06; 95% CI = 0.84 to 1.34; *P *= 0.63 and OS: HR = 1.07; 95% CI = 0.88 to 1.32; *P *= 0.49). Individuals who were heterozygous at more than one site were excluded as the haplotype arrangements of these variants (whether they were arranged in cis or in trans) could not be unequivocally determined.

We did find some evidence for association of one PM variant, CYP2D6*6b/6c, with reduced BCSS and OS in women treated with tamoxifen. Carriers of the minor allele had poorer survival (BCSS: HR = 1.95; 95% CI = 1.12 to 3.40; *P *= 0.02 and OS: HR = 1.91; 95% CI = 1.18 to 3.11; *P *= 0.01). As expected for such a rare variant (MAF = 0.01), no patients in this study were homozygous for the minor allele. This association with decreased BCSS becomes non-significant when restricted to ER-positive patients who received tamoxifen and after adjustment for the known prognostic factors: stage, grade, tumour size, nodal status, surgery, and chemotherapy (Table 2; HR = 1.80; 95% CI = 0.56 to 5.80; *P *= 0.33). None of the other functional or tagSNPs were associated with BCSS after adjustment for ER.

Homozygote deletions CY2D6*5/*5, and a combined heterozygotes (CY2D6*1/*5 and CY2D6*1/*2×*1) and common allele homozygotes (CY2D6*1/*1) group were defined. Using our criteria, there were 40 CYP2D6*UM in our study population, giving a MAF of 0.08. This is higher than the previous reports of homozygote CYP2D6*UM with a MAF of 0.02. During the analysis, the association of CYP2D6*UM with BCSS was assessed for CYP2D6*UM with a MAF of 0.08 and also CYP2D6*UM with a MAF of 0.02 (previously reported MAF). There was no significant association with either BCSS or OS in either case.

Regardless of genotype, the use of CYP2D6 inhibitors (e.g. fluoxetine, cimetidine) did not affect BCSS or OS for patients either receiving tamoxifen treatment or not receiving tamoxifen treatment. This was true for all functional SNPs and tagSNPs.

Some of the studies reporting an association between *CYP2D6 *PM variants and endpoints such as DFS or time to relapse (TTR), had restricted their analysis to post-menopausal women, who were ER positive but had not received chemotherapy and who were treated with tamoxifen. Unadjusted sub-group analysis of cases restricted to these criteria in our study showed no association with BCSS or OS for either individual variants or for combined PM/IM groups. Similarly, analysis of pre-menopausal cases also showed no association with BCSS or OS for either individual variants or for combined PM/IM groups.

Genotyping this region of the genome is complicated by the presence of pseudogenes. There is one variant for which the genotype deviated significantly from HWE CYP2D6*41 (P_HWE _= 5.92 × 10^-6^). Large deviations in HWE may be indicative of poor genotyping specificity as a result of cross-hybridisation of the assay with pseudogene sequences. Potential misclassification of alleles means that reported associations with these variants should be treated with caution.

## Discussion

This large study has investigated the association between germ-line variation in *CYP2D6 *variants and BCSS in 3,155 patients treated with tamoxifen and 3,485 patients who did not receive tamoxifen. The main strengths of the study are the sample size, the comprehensive evaluation of *CYP2D6 *variation, the systematic follow-up and the high quality of genotyping assays using DNA extracted from blood samples.

Our results suggest that just one putative PM variant, CYP2D6*6 (1976g>a variant), may be associated with decreased BCSS. This association cannot be considered definitive because it is not highly statistically significant. Wacholder and colleagues [48] define the false positive reporting probability, which provides a useful framework to evaluate the importance of any statistical association that is dependent on the prior probability of association and the power to detect that association. Although there is good pharmacokinetic evidence to support the existence of PM and IM *CYP2D6 *variants, the evidence linking these variants to clinical outcome in tamoxifen-treated breast cancer patients is much weaker. Assuming a prior probability of association of 1 in 10 for a hazard ratio of 1.5 the association between CYP2D6*6 and outcome has a 50% chance of being a false positive. For a prior probability of 1 in 100, the false positive probability is over 90%. No other tagSNPs or functional SNPs showed any association with BCSS. Similarly, when considering the combinations of PM/IM variants the results do not support the hypothesis that putative PM or IM variants, in general are associated with poorer survival. Our data support the conclusion of Lash and colleagues [27,28] that *CYP2D6 *variants are not significantly associated with outcome in women with breast cancer treated with adjuvant tamoxifen.

Supplementary table S8 in Additional file 1 summarises the recent key studies, their results and methods. These studies are highly heterogeneous both in methodology and results. Previous studies have reported hazard ratios of over two for relapse free survival for different genotypes. Assuming a slightly weaker association with BCSS (for example, HR = 1.85), we have 65% power to detect a difference in a sub-group (defined by genotype) of 1% of the patient population and over 90% power to detect that difference in a subgroup of 2% of the population [see Supplementary table S3 in Additional file 1]. The PM/IM1 group comprises 8.95% of patients. We have over 90% power to detect a HR of 1.85 in this group. Thus, our study is extremely well powered to detect the effects that have previously been reported. It is possible that one or more of these variants are associated with a specific sub-group of cases, but the number of ways of classifying tumours is large and such a specific sub-group effect can rarely be excluded. Furthermore, there are no published data suggesting that an association would be limited to a more specific sub-group of cases.

We detected a higher frequency of CYP2D6*UM in this study than has been previously reported. This may be the true value for our population, however, because we had no homozygote CYP2D6*UM (CY2D6*2 × *1/*2 × *1) positive control, it is also possible that there has been some misclassification. Some of the heterozygote CYP2D6*UM may have been included in the homozygote CYP2D6*UM group. However, to assess the potential effect of this on association with BCSS and OS, the analysis was performed for CYP2D6*UM MAF of 0.08 and also CYP2D6*UM MAF of 0.02 (previously reported MAF). Patients with the UM variant, which is predicted to generate increased plasma concentrations of activate metabolites, did not show any evidence of improved BCSS or OS relative to EM, irrespective of whether the MAF was 0.08 or 0.02.

The presence of the two highly homologous pseudogenes complicates the genotyping of *CYP2D6 *gene variants [30,12]. For the CYP2D6*4 variant, a nested or long range PCR approach is needed to ensure specificity during genotyping. We used a nested PCR approach and assessed the specificity of the primers by sequencing. The use of inadequately specific primers may in some variants lead to unreliable genotyping classification and subsequent clinical associations. HWE may be used as an aid to assess genotyping quality [31]. Examination of published data shows that in some studies, the genotype distribution of certain variants deviate significantly from HWE, for example, Goetz and colleagues [13] (CYP2D6*4 P_HWE _= 2.4 × 10^-4^) and Schroth and colleagues [21] (CYP2D6*4 P_HWE _= 0.009 and CYP2D6*10 P_HWE _= 1.7 × 10^-7^), suggesting that genotype classification of this region of the genome is problematic, meaning that any clinical associations found should be interpreted with care. Such deviation from HWE is unlikely to be due simply to population admixture or the fact that it is a patient cohort.

There may be some concern that the use of prevalent cases is problematic in any time-to-event analysis even though this was taken into account in the analysis. The analysis was, therefore, repeated on the subset of the subjects ascertained as incident cases. There was no substantial difference in the HR estimates for CYP2D6*6. The results showed CYP2D6*6 P = 0.03, HR = 2.09 and 95% CI = 1.07 to 4.07 for the incident cases only analysis, in comparison to *P *= 0.02, HR = 1.95 and 95% CI = 1.12 to 3.40 when the analysis was completed using both incident and prevalent cases combined. The results for CYP2D6*4 for the incident cases again showed no significant association with BCSS (*P *= 0.4, HR = 0.90, 95% CI = 0.70 to 1.15).

We have examined all the available evidence on *CYP2D6 *variants and outcome for breast cancer patients treated with adjuvant tamoxifen including our own data presented here. Many studies have used PFS, DFS, or TTR as the clinical endpoint of choice. It may be argued that the effects of *CYP2D6 *genotypes are specific to relapse and not death. However, DFS and PFS data are not available for the patients in our study. Nevertheless, it is very unlikely that any of these variants have a large effect on BCSS with over seven years median follow-up on patients treated with tamoxifen. Furthermore, it could be argued that a difference in PFS is not clinically useful if that does not result in an improvement in BCSS [49].

Other potential weaknesses of our study also need to be considered. Detailed information on patient compliance and length of tamoxifen therapy are currently unavailable. However, given that the HR for CYP2D6*4 was very close to unity (HR = 1.01), any true effect limited to compliers would have had to have been balanced by the opposite effect of greater magnitude in non-compliers, which seems unlikely. Our evaluation of functional variants was not comprehensive. We were not able to assess CYP2D6*3 due to failure of manufacture of an adequate Taqman assay. This would have resulted in some misclassification of PM patients as EM, but this misclassification will have been very small as CYP2D6*3 is found in 1 to 4% of the Caucasian population [50]. Furthermore, only 3 of the 12 published studies shown in Supplementary table S8 in Additional file 1 investigated this variant and none of these studies reported a significant association for this variant individually.

Our case series is unselected for family history and does not have any over-representation of hereditary breast cancer. Our study population represents the heterogeneous group of patients for whom *CYP2D6 *testing might be used in practice. Our patients include both pre- and post-menopausal cases; however, there is little evidence to suggest that women with ER-positive breast cancer respond differently to tamoxifen according to menopausal status. The Early Breast Trialists Overview [2] report a similar effect of tamoxifen in women aged less than 50 years old as those over 50 years of age.

## Conclusions

We conclude that the evidence for variation in the efficacy of tamoxifen treatment by *CYP2D6 *PM/IM functional status is at best limited. It is therefore premature to use *CYP2D6 *testing to guide therapy with tamoxifen.

## Abbreviations

AI: aromatase inhibitor; BCSS: breast cancer specific survival; CI: confidence interval; CYP2D6: cytochrome P450 2D6; DFS: disease free survival; ECRIC: Eastern Cancer Registration and Information Centre; EM: extensive metaboliser; ER: oestrogen receptor; FDA: Food and Drug Administration; HR: hazard ratio; HWE: Hardy Weinberg Equilibrium; IM: intermediate metaboliser; MAF: minor allele frequency; OS: overall survival; PCR: polymerase chain reaction; PFS: progression free survival; PM: poor metaboliser; SNP: single nucleotide polymorphism; TTR: time to response; UM: ultra-metaboliser.

## Competing interests

The authors declare that they have no competing interests.

## Authors' contributions

JEA and CC conceived the study idea. JEA, CC, PDPP and AD designed the study. JEA and MM planned and performed the Taqman PCR and real-time PCR. JEA, SI, MS and DG collected the data. RP, BK, CB and CL performed the nested PCR, sequencing and gel electrophoresis. JEA, KD and PDPP planned and performed the analyses. JEA reviewed the literature and drafted the manuscript. CC, PDPP, AD and HME edited the manuscript.

## Supplementary Material

Additional file 1 **Supplementary tables S1 to S6 and S8**. Supplementary table S1: CYP2D6 inhibitors. A table containing the generic and trade names of CYP2D6 inhibitor drugs used by patients in the study. Supplementary table S2: All Functional and TagSNPs attempted. A table containing all the functional and tagSNPs attempted including those which failed. Supplementary table S3: Power calculations. Tabulated summary of power available in this study for each variant. Supplementary table S4: Genotype and allele frequencies. Tabulated summary of genotype and allele frequencies for each variant. Also the number of deaths (all-cause) in tamoxifen-treated patients. Supplementary table S5: Genotype frequencies and results of unadjusted and adjusted Cox regression analysis of common (tagSNPs) and functional polymorphisms and overall survival. Supplementary table S6: Full unadjusted CYP2D6 survival analysis independent of tamoxifen status. Tabulated summary of the full unadjusted CYP2D6 survival analysis independent of tamoxifen status. Supplementary table S8: Summary of previous studies. Tabulated summary of previous studies relating to CYP2D6 variants, clinical response and tamoxifen. Click here for file

Additional file 2 **Supplementary figure S1: Sequence alignment**. The figure and supporting legends showing the primers and sequences for CYP2D6 and its known pseudogenes. Click here for file

Additional file 3 **Supplementary table S7: Adherence to REMARK criteria**. Details of the REMARK criteria and how we have adhered to it. Click here for file
