# Supplementary material for: CYP2D6 gene variants: association with breast cancer specific survival in a cohort of breast cancer patients from the United Kingdom treated with adjuvant tamoxifen
Source: Breast Cancer Res. 2010 Aug 23;12(4):R64. doi: 10.1186/bcr2629 (PMC2949659; doi:10.1186/bcr2629)
Supplement: Additional file 2 — Supplementary figure S1: Sequence alignment. The figure and supporting legends showing the primers and sequences for CYP2D6 and its known pseudogenes. [file bcr2629-S2.DOC]

**Legends for Supplementary Figure 1**

**Title: Primers used for CYP2D6*4 nested PCR and their priming sites in pseudo-genes:**

**Shaded regions indicate nucleotide base differences between CYP2D6 (in bold) and pseudogenes**

**Chromosome positions are given according to human chromosome 22 reference sequence assembly from Annotated Genome Build 37.1 (NC_000022.10)**

**Chromosome position of SNP CYP2D6*4: 42538148**

**Supplementary Figure 1**
