# Supplementary material for: CYP2D6 gene variants: association with breast cancer specific survival in a cohort of breast cancer patients from the United Kingdom treated with adjuvant tamoxifen
Source: Breast Cancer Res. 2010 Aug 23;12(4):R64. doi: 10.1186/bcr2629 (PMC2949659; doi:10.1186/bcr2629)
Supplement: Additional file 3 — Supplementary table S7: Adherence to REMARK criteria. Details of the REMARK criteria and how we have adhered to it. [file bcr2629-S3.DOC]

**Supplementary Table 7: Adherence to REMARK (42) criteria**

| **Reporting recommendations for REMARK** | **How criteria are fulfilled** |
| --- | --- |
| **Introduction** |  |
| 1. State the marker examined, the study objectives, and any pre-specified hypotheses | Clear statement of hypothesis and study objectives: p3-5 |
| **Materials and Methods** |  |
| *Patients* |  |
| 1. Describe the characteristics of study patients, including their source and inclusion and exclusion criteria. | Table 1 describes characteristics of study patients.  Population based cohort study, eligibility and study population details p6-7 |
| 2.Describe treatments received and how chosen | Rule-based treatment decisions and details p6 |
| *Specimen Characteristics* |  |
| 1. Describe type of biological material used and methods of preservation and storage | Whole blood samples used p6. Prior to extraction the blood is stored at -80C. Post-extraction DNA is stored in trisEDTA at -30C to -40C. |
| 2. Specify the assay used and provide (or reference) a detailed protocol, including specific reagents or kits used, quality control procedures, reproducibility assessments, quantitation methods, and scoring and reporting protocols. Specify whether and how assays were performed blinded to the study endpoint. | p8-10 give details of assays and protocols used.  Assays were performed blinded to the study endpoint. |
| *Study Design* |  |
| 1. State the method of case, selection including whether prospective or retrospective and whether stratification or matching was used. Specify the time period from which cases were taken, the end of the follow-up period, and the median follow-up time. | Details of case selection and time period p6-7 |
| 2. Precisely define all clinical endpoints examined. | P6-11 give details of endpoints. |
| 3. List of all candidate variables initially examined for inclusion in models. | P8-10 and Supplementary table 2 gives details of all *CYP2D6* variants. |
| 4. Give rationale for sample size; if the study was designed to detect a specified effect size, give the target power and effect size. | The power of this study was dependent upon allele frequency and genetic mode of action of the variants studied. |
| *Statistical analysis methods* |  |
| 5. Specify all statistical methods, including details of any variable selection procedures and other model-building issues, how model assumptions were verified, and how missing data were handled. | P10-11 |
| 6. Clarify how marker values were handled in the analysis. | P7-11 |
| **Results** |  |
| *Data* |  |
| 1. Describe the flow of patients through the study, including the number of patients included in each stage of the analysis and reasons for drop out. Specifically, report the number of patients and the number of events. | P6-7 describes the flow of patients. This was not a staged analysis. Supplementary tables 1-5. |
| 2. Report distributions of basic demographic characteristics, standard prognostics variables | Table 1 gives breast cancer patient characteristics. Standard prognostic indicators used in adjusted analysis are listed on p13. |

**Supplementary Table 7(continued): Adherence to REMARK (42) criteria**

| *Analysis and presentation* |  |
| --- | --- |
| 1. Show the relation to standard prognostics variables. | Table 2 gives both adjusted and unadjusted results |
| 2. Present univariate analyses showing the relation between between the marker and outcome, with the estimated effect (e.g. hazard ratio and survival probability). Preferably provide similar analyses for all other variables being analysed. For the effect of marker (variant in this case) on a time-to-event outcome, a Kaplan-Meier plot is recommended. | Table 2 gives both adjusted and unadjusted results. |
| 3. For key multivariable analyses, report estimated effects (e.g. hazard ratio) with confidence intervals for the variant and, at least for the final model, all other variables in the model. | Table 2 gives both adjusted and unadjusted results |
| 4. Among reported results, provide estimated effects with confidence intervals from an analysis in which the marker and standard prognostic variables are included regardless of their statistical significance. | Table 2 gives both adjusted and unadjusted results  Table 3 gives unadjusted results independent of tamoxifen treatment |
| 5. If done, report results of further investigations, such as checking assumptions, sensitivity analyses, and internal validation. | Not applicable |
| **Discussion** |  |
| 1. Interpret the results in the context of the pre-specified hypothesis and other relevant studies; include a discussion of limitations of the study. | p15-18, Supplementary table 8 |
| 2. Discuss implications for future research and  clinical value. | p15-18 |
